# Exploring the Role of External Beam Radiation Therapy in Osteosarcoma Treatment: Impact of Diagnostic Imaging Delays and Innovative Techniques

**DOI:** 10.7759/cureus.37442

**Published:** 2023-04-11

**Authors:** Alec B Landau, Vivian S Zhu, Akshay J Reddy, Chetan Yarlagadda, Matthew Corsi, Levi M Travis, Mohamed Abutineh, Ali Idriss, Rakesh Patel

**Affiliations:** 1 Health Sciences, California Northstate University, Rancho Cordova, USA; 2 Ophthalmology, California University of Science and Medicine, Colton, USA; 3 Internal Medicine, Florida Atlantic University Charles E. Schmidt College of Medicine, Boca Raton, USA; 4 Orthopaedics, Wayne State School of Medicine, Detroit, USA; 5 Medicine, University of Miami Miller School of Medicine, Miami, USA; 6 Internal Medicine, Edward Via College of Osteopathic Medicine, Spartanburg, USA; 7 Anesthesiology, Florida Atlantic University Charles E. Schmidt College of Medicine, Boca Raton, USA; 8 Internal Medicine, East Tennessee State University - Quillen College of Medicine, Johnson City, USA

**Keywords:** radiology review articles, radiology research, bone cancer, radiation therapy, cancer imaging, osteosarcoma, external beam radiation therapy

## Abstract

Osteosarcomas are a type of bone cancer that typically affect young adults, often in the bones of the arms and legs. To treat osteosarcoma, doctors typically use a combination of chemotherapy, radiotherapy, and surgery, with External Beam Radiation Therapy (EBRT) being the most commonly used form of radiotherapy. EBRT involves directing high-energy photons, X-rays, gamma rays, protons, and electrons at the tumor to induce cancer cell death. Additionally, healthcare providers use imaging techniques to monitor treatment success. This literature review aims to explore the relationship between osteosarcomas and EBRT, investigate the impact of the delayed diagnosis on survival rates, and examine the effectiveness of innovative uses of EBRT for treating osteosarcomas in unusual locations using comprehensive diagnostic techniques. To achieve these objectives, the review examines case studies and literary analyses and categorizes them based on the delay between symptom onset and diagnosis. The null hypothesis is that the presence or absence of a delay in diagnosis does not significantly impact outcomes for the "Delay" category. A lack of delay results in a more favorable outcome in the "Lack of Delay" category. However, the data and statistical results suggest that additional follow-up care in patients with rare or commonly recurring cancers could benefit outcomes. It is important to note that due to the rarity of osteosarcoma with EBRT, the small sample size in the studies warrants further investigation. Interestingly, many patients presented with head and neck tumors despite the most common location of osteosarcoma being in the long bones.

## Introduction and background

Osteosarcoma, also known as osteogenic sarcoma, is the most common type of cancer that originates in the bone and is often found in the extremities [1]. Osteosarcoma has an incidence rate of 3.4 cases per million people worldwide and roughly 1,000 new cases yearly in the United States [1]. The two categories of osteosarcomas, primary and secondary, originate in bone cells-however, primary osteosarcomas present as a solid tumor due to an abnormality in bone development. At the same time, secondary osteosarcoma is also a solid tumor, but instead due to another underlying bone condition [2]. Secondary osteosarcoma is thought to be developed with the cumulative effects of radiation exposure on bone cells [2]. Due to these differences, primary osteosarcoma is typically found in developing adolescents, and in contrast, secondary osteosarcoma is generally found in adults with fully developed bones [2]. The average age at which the primary form of osteosarcoma is diagnosed is at age 15; boys and girls are equally likely to develop osteosarcoma during puberty, specifically in areas where bone cells proliferate quickly [3]. Therefore, the most common sites to find tumors are in the proximal humerus, the proximal tibia and distal femur, and bones around the knee [1]. The exact cause of osteosarcoma development is unknown, but risks can be associated due to environmental and genetic factors, including DNA modifications acquired before or after birth.

Some risk factors include teenage growth spurts, certain benign bone disorders, high radiation exposure at a young age, and being tall for a particular age [4]. In addition, studies have recently examined genetics' contribution to osteosarcoma development [5]. The fact that a quarter of the study participants with a cancer diagnosis carried mutations associated with an increased risk of cancer suggests that genetic testing may be helpful in the diagnosis process [5]. Healthcare providers most commonly treat osteosarcoma with surgery, chemotherapy, and radiation therapy after diagnosis. The surgery physically removes the tumor, and chemotherapy promotes apoptosis among the remaining cancer cells to prevent further proliferation [6]. This paper will focus on the most common type of radiation therapy utilized for osteosarcoma treatment: External Beam Radiation Therapy (EBRT) [7]. Due to its precision and efficiency, it serves as the default treatment for various types of common cancers, such as lung cancer, breast cancer, melanoma, uterine cancer, and prostate cancer [8]. Although healthcare providers can use EBRT as a complementary treatment to other therapies, such as chemotherapy, they can also utilize it to shrink tumors before surgery and provide palliative care by destroying tumors that interfere with the patient's quality of life [8]. Unfortunately, even though EBRT can target cancer cells and minimize damage to healthy cells, it inevitably causes the death of healthy cells, resulting in the side effects of radiation therapy such as nausea, vomiting, headaches, hair loss, incontinence, and tenderness at the treatment site [8]. While treatment options for secondary osteosarcoma are similar to those for primary osteosarcoma, it is crucial to note that the prognosis for secondary osteosarcoma is generally worse, and treatment outcomes may be more challenging to predict [2].

This paper examines the impact of the time between osteosarcoma symptom onset and diagnosis on the effectiveness of EBRT treatment. Typically, diagnosing osteosarcoma requires a combination of imaging techniques, medical history, and physical exams. Early detection through preventative screening and prompt follow-up treatment with EBRT can significantly improve treatment outcomes. However, many barriers, such as limited awareness, cost, and access, can hinder early intervention. Surprisingly, researchers have yet to investigate the effect of delayed diagnosis on EBRT treatment outcomes compared to those who receive prompt diagnosis and treatment. Therefore, proving a definitive relationship between a lack of delay in diagnosis and positive health outcomes would provide a valuable indicator that the added expense would be worth the investment.

EBRT mechanisms for inducing apoptosis in cancer cells

EBRT consists of utilizing a linear accelerator to speed up charged photons, x-rays, gamma rays, protons, and electrons and deliver these beams directly to the tumor with precise doses of radiation daily over weeks to months [8]. EBRT induces apoptosis in cancer cells by damaging their Deoxyribonucleic acid (DNA) [9]. When the radiation beam passes through the body, it damages the DNA in the cells it encounters [10]. Severe damage to DNA caused by X-ray irradiation can trigger a series of molecular events that may result in cell death through apoptosis or other factors, such as necrosis or autophagic cell death [10]. Apoptosis is a complex process in which a cell undergoes a series of changes that ultimately lead to its programmed cell death. These changes include shrinkage of the cytoplasm, condensation of the nucleus, and fragmentation of the DNA. Several signaling pathways, such as the p53 and intrinsic apoptotic pathways, respond to DNA damage, which activates apoptosis. During apoptosis, enzymes called caspases are activated, leading to the breakdown of the cell into smaller fragments that the immune system can safely remove. This process helps to prevent the spread of cancer and reduce the size of the tumor. Understanding that EBRT kills cancer cells and affects normal healthy cells in the treated area is essential. Fortunately, normal cells can repair their DNA and recover from radiation exposure, while cancer cells often lack this ability, making them more vulnerable to the effects of radiation therapy.

## Review

Methods

**Figure 1:**
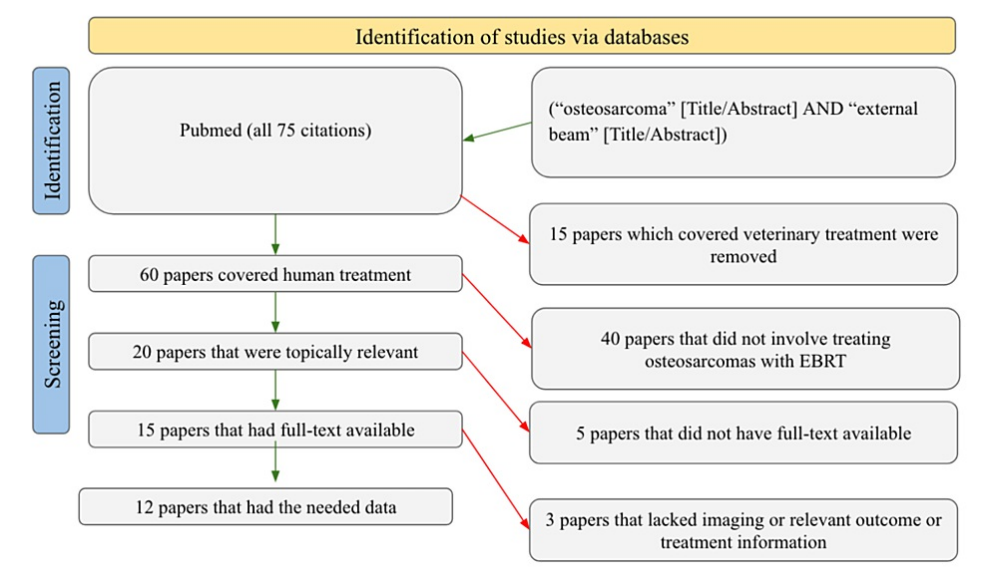
PRISMA diagram illustrating the article filtration process. PRISMA: Preferred Reporting Items for Systematic Reviews and Meta-Analyses.

We searched PubMed for studies about using EBRT to treat osteosarcomas across all timeframes, resulting in 75 papers. Of these, 60 papers focused on treating humans, 20 were topically relevant, 15 had full text available, and only 12 papers included the necessary data for analysis. In investigating these 12 papers, the authors gathered information on the imaging technique used, whether EBRT was used, whether other treatments were used in conjunction, the sample size, length of treatment, location of the tumor, outcome, and whether there was a delay in diagnosis relative to the outcome. Studies that needed more data to overlap with at least three categories mentioned above were eliminated. All articles meeting the criteria were retained, and each article was evaluated by every author, offering a comprehensive analysis of each paper. Figure 1 illustrates our filtering procedure according to the Preferred Reporting Items for Systematic Reviews and Meta-Analyses (PRISMA) guidelines used in this study. In addition, a one-sample t-test was performed to independently compare the patient outcomes within each of the "Delay" and "No Delay" categories. All statistical analyses were performed using Statskingdom.

Impact of Imaging

Synchronous metastases, or metastatic tumors found upon diagnosis with the primary tumor, differ in rates depending on diagnostic delay, access to diagnostic and preventive screening, and location. On average, synchronous metastases are detected in 10-30% of osteosarcoma patients in high-income countries and about 40% in low-income countries due to modern imaging techniques or lack thereof [9].

In addition, research shows that 80% of widespread metastases resulting from osteosarcoma are found in the lungs, while the remaining spread throughout the body, including in the same type of bone found elsewhere. Studies using meta-analysis have indicated that osteosarcoma can recur in 30-50% of patients with localized tumors and in up to 80% of patients with metastatic disease. Several known mechanisms promote metastasis, including tumor heterogeneity, gene downregulation, and the involvement of tumor-associated macrophages.

Osteosarcoma can present with a range of symptoms, including a warm, painless lump that can be palpated through the skin, bone fractures with normal movement, limited range of motion in the affected limb, and increased pain during activity [3]. To diagnose osteosarcoma, a variety of tests are typically used, including X-rays to visualize internal tissue, bone scans to identify sources of inflammation and pain, Magnetic resonance imaging (MRI) to detect the presence of cancerous masses, Computed tomography (CT) scans to reveal details of internal structures, and Positron emission tomography (PET) scans to track glucose uptake through radioactive tracers [4]. Other tests may include Complete blood count (CBC) to measure white blood cell counts, biopsies to detect metastasis, and Alkaline phosphatase (ALP) tests to gauge bone activity, as elevated ALP levels may indicate the presence of bone cancer. However, it is essential to note that not all individuals with osteosarcoma will have elevated ALP levels [11]. Furthermore, genetic testing is increasingly used as a screening tool for children with inherited genes predisposing them to cancer development.

In order to thoroughly investigate the relationship between osteosarcomas and EBRT, we conducted a comprehensive literature review incorporating a diverse range of study types, including case studies, chart reviews, and experimental trials. By incorporating different study types, we obtained a more comprehensive and nuanced understanding of the subject matter. Case studies and chart reviews allowed us to thoroughly analyze individual patient cases, while experimental trials provided insights into the effectiveness of various treatment approaches under controlled conditions. Through this approach, we were able to examine the impact of imaging on patient outcomes in osteosarcoma cases.

To measure the impact of imaging, we identified 12 articles that met our inclusion criteria. This information is displayed in Table 1 [12-23]. Physicians treating patients with osteosarcoma utilized EBRT in various manners with other treatments in conjunction to evaluate patient outcomes. In 9 out of 12 studies, multiple imaging modalities were used. Studies have shown that using multiple imaging modalities increases accuracy and sensitivity in diagnosing and staging diseases, improves treatment planning, and enhances patient outcomes [24]. This is because utilizing multiple imaging modalities allows for obtaining complementary information from each modality, providing a more comprehensive understanding of the evaluated disease or condition [24]. In the table, we categorized patients as having a "Delay" or "No Delay" based on whether there was a time difference between the onset of cancer and the diagnosis that led to treatment. "No Delay" implies that patients immediately receive diagnostic treatment and a diagnosis after the presence of symptoms. If there was any time difference between the onset of symptoms and the official diagnosis, the patient was classified in the "Delay" category. If a patient had a "Delay" but the time difference did not impact the outcome, or if there was insufficient information in the literature to conclude, we marked the result as "N/a" in the table.

**Table 1:** Current applications of EBRT treating osteosarcomas with a diagnostic imaging perspective. SPECT: Single-photon emission computed tomography; ^186^Re-HEDP: 186Re(Sn) hydroxy ethylidene bisphosphonate; CECT: Contrast-enhanced computed tomography; CPAP: Continuous Positive Airway Pressure; SBRT: Stereotactic body radiation therapy; EBRT: External Beam Radiation Therapy

Author (year)	Imaging Technique	Treatments Used In Conjunction With EBRT	Sample Size	Length of Treatment	Location of Tumor	Outcome	Delay of Diagnosis Relation to Outcome?
Syed et al. (2006) [12]	CT transaxial slice + SPECT whole body scan	^186^Re-HEDP	13	2-4 months	Lung-related (12), Scapula-related (1)	Death (12), Survival (1)	N/a
Papandreou et al. (2010) [13]	CT	Transurethral Resection	1	Four months	Urinary Bladder	Delayed Death, Poor Quality of Life	Delay
Morris et al. (2006) [14]	CT+MRI	N/a	1	N/a	Eyes	Death	Delay
Machak et al. (2003) [15]	CT+MRI+Planar Scintigraphy	Various Chemotherapies	31	3-6 months	Extremities	Death (12), Survival (19)	Delay
Kallianpur et al. (2013) [16]	CECT thoracoabdominal +whole body bone scan+ Photomicrography	Mastectomy, adjuvant Adriamycin + Cisplatin	1	4 months	Breast	Survival	No Delay
Jimenez et al. (2020) [17]	MRI	N/a	20	N/a	Craniofacial	Death (12), Survival (8)	Delay
Imhoff et al. (1997) [18]	CT+MRI	Adjuvant Chemotherapy	1	N/a	Orbit	Death	Delay
Douglas et al. (1997) [19]	CT+ Radiogram+Ultrasound	Tumour Resection+ CPAP Ventilator	1	N/a	Sternum	Survival	No Delay
DeLaney et al. (2005) [20]	CT+MRI	Gross Total Tumor Resection (27), Subtotal Resection (9), Biopsy Only (5)	41	N/a	Head/face/skull (17, Extremity (8), Spine (8), Pelvis (7), Trunk (1)	Death (11), Survival (30)	N/a
Couldwell et al. (1997) [21]	Bone Scan+ Chest and Abdominal CT+ MRI	Formerly Completed Left Fronto-Temporal Craniotomy, Carmustine	1	5 months	Meninges	Death	No Delay
Bhatt et al. (2014) [22]	CT	Prior near-total laryngectomy	1	N/a	Larynx	Survival	No Delay
Anderson et al. (2020) [23]	CT+PET+SPECT	Radium-223+SBRT	15	3 to 15 Months	N/a	Death (14), Survival (1)	N/a

EBRT compared to alternative radiation therapies

In contrast to EBRT, other forms of radiation therapy include brachytherapy, where the radioactive source is placed directly into or near the cancer cells, and internal radiation therapy, where radioactive material is placed inside the body [25]. EBRT has several advantages over these other forms of radiation therapy, including a higher dose of radiation delivered, a lower risk of radiation exposure to surrounding healthy tissues, and a more controlled radiation delivery [25]. Furthermore, the local control rate for EBRT varies depending on the cancer type and stage but can range from roughly 50% to over 90% [25].

However, one major problem that can affect the effectiveness of EBRT in treating osteosarcomas is delays in imaging diagnosis. Osteosarcomas are often challenging to diagnose due to their location within the bone and tendency to mimic other conditions [12]. Delays in imaging diagnosis can cause a delay in starting EBRT, which can reduce its effectiveness in treating cancer [17]. Cancer cells can continue to proliferate and spread during the delay, making them more resistant to radiation therapy and increasing the risk of severe side effects, such as damage to healthy tissues or organs [17].

Maximizing local control with EBRT

According to current medical literature, EBRT is best used with other osteosarcoma treatments, such as surgery and chemotherapy [26]. However, as a single modality, radiation therapy is not successful in either reliably controlling the primary tumor or preventing the appearance of lung metastases [15]. 

The dose and fractionation of EBRT can vary depending on the stage and location of the osteosarcoma [20]. A high radiation dose is typically delivered in fewer fractions for more aggressive tumors, while a lower dose delivered over more fractions is often used for less aggressive tumors [20]. The use of image-guided radiation therapy (IGRT) and intensity-modulated radiation therapy (IMRT) has improved the delivery of EBRT for osteosarcoma [27]. IGRT uses imaging techniques to target the radiation to the tumor accurately. In contrast, IMRT uses advanced technology to deliver a more precise radiation dose to the tumor while sparing normal tissues [27].

Age is a critical factor in the outcomes of EBRT treatment. For instance, a study showed that patients with Retinoblastoma who received EBRT before 12 months of age were at a higher risk of developing craniofacial secondary primary tumors than those treated at an older age [17]. Nevertheless, EBRT remains a viable option for treating metastatic or unresectable recurrent lesions, even at high doses in a palliative setting [28].

Impact of delayed imaging diagnosis on survival rates

We divided the studies and analyses that met the criteria into two categories based on the time between symptom presentation and diagnosis and analyzed them. The null hypothesis, stating that the presence or absence of a delay in diagnosis has no effect, was not rejected for the "Delay" category. However, for the "Lack of Delay" category, the P-value was less than 0.05, indicating that the null hypothesis was rejected.

**Figure 2:**
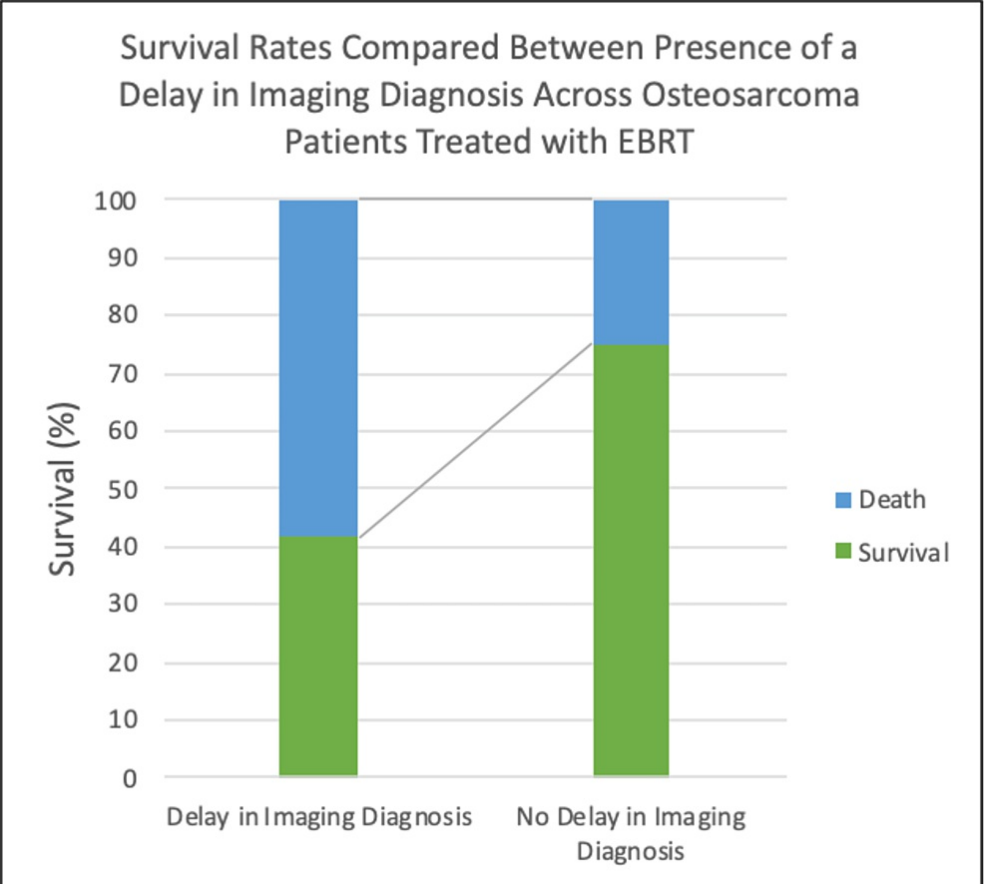
Visualizing Survival Rates. A bar graph visualizes the survival rates between osteosarcoma patients treated with EBRT with a delay in imaging diagnosis in contrast to those without a delay in imaging diagnosis.

Furthermore, the T-test results showed a value of approximately 3 for the "No Delay" category and roughly 1.6 for the "Delay" category, indicating a strong association between the lack of delay and positive outcomes. Interestingly, the study found that a delay in diagnosis did not affect the outcome, which aligns with current follow-up practices. However, the two groups showed a significant survival rate difference, as shown in Table 2 and Figure 2. These findings suggest that patients with rare or frequently recurring cancers may benefit from additional follow-up care to improve their outcomes.

**Table 2:** Statistical Analysis. A table is shown above to organize the statistical results for the survival rate, P-value and resulting confirmation or denial of statistical significance, and T-test value in the “Delay” and “No Delay” categories.

	Delay in Imaging Diagnosis	No Delay in Imaging Diagnosis
Overall Survival Percentage (%)	41.67	75.00
P-Test Value	0.0945	0.02883
T-Test Value	1.5811	3

Real-world implications: Optimizing follow-up care for high-risk patients

Diagnostic imaging plays a critical role in identifying cancer at an early stage for high-risk patient populations, including those with recurring cancers and a family history of cancer. By providing essential information about the progression and stage of cancer, imaging can help healthcare providers make informed treatment decisions and empower patients to take an active role in their care.

While diagnostic imaging can have potential downsides, such as radiation exposure and increased costs, the benefits of early detection and effective treatment outweigh these risks for many patients. Moreover, ongoing monitoring of treatment effectiveness and detecting changes in the condition allows healthcare providers to intervene and adjust treatment plans as needed.

Therefore, healthcare providers must carefully consider the benefits and risks of diagnostic imaging to deliver optimal care for their patients. With proper use, imaging is a valuable tool in improving outcomes and advancing patient-centered care.

Areas for further investigation and current constraints

Despite selecting studies with innovative approaches to EBRT and unique locations of osteosarcomas, our sample size was unavoidably limited. Although most studies had only one sample size with minimal cases across years or decades, we identified a roughly 34% increase in survival rates for those who received a prompt diagnosis. This emphasizes the importance of follow-up care and imaging for specific patient populations, though more research is needed to verify these findings. It is important to note that certain quality studies were excluded due to the need for more precise information on treatment length, diagnosis, and patient care. As this paper is the first investigation into survival rates from a presence or lack of a delay from the onset of symptoms to imaging diagnosis, there is an inherent lack of benchmarks or community guidelines. Nonetheless, we remain optimistic about the potential for future research to confirm our findings and explore the most effective diagnostic imaging techniques. By prioritizing early detection and intervention, we can improve outcomes for individuals affected by osteosarcoma and other recurring cancers.

## Conclusions

This literature review offers valuable insights into the interplay between osteosarcomas and EBRT. Osteosarcoma, a form of bone cancer primarily affecting young adults and teenagers, is commonly treated with EBRT, which is most effective when initiated promptly. The study investigated the impact of delayed diagnosis of osteosarcoma on the outcomes of EBRT treatment. The results of this study are like a ray of hope for cancer survivors, indicating that prompt diagnosis and follow-up care can significantly enhance the effectiveness of EBRT treatment. On the other hand, delayed diagnosis may hinder treatment initiation, causing reduced treatment efficacy. To treat osteosarcoma, a combination of surgery, chemotherapy, and EBRT is typically used, with the most effective dosage and fractionation depending on the stage of the disease and patient-specific factors. However, osteosarcoma screening is limited due to low awareness, high costs, and lack of accessibility. Therefore, this study highlights the importance of further research to uncover the genetic factors underlying osteosarcoma development and the potential of genetic testing in diagnosis.

This study provides critical insights into the diagnosis and treatment of osteosarcoma through a comprehensive and critical evaluation of the existing literature, which can inform future research and clinical decision-making. Future research will play a crucial role in unraveling the genetic underpinnings of osteosarcoma and enhancing diagnostic methods, ultimately benefiting patients in need.

## References

[1] (2023). What is Osteosarcoma?. https://www.cancer.org/cancer/osteosarcoma/about/what-is-osteosarcoma.html.

[2] (2023). Types of Osteosarcoma - Moffitt Cancer Centeer. https://moffitt.org/cancers/osteosarcoma/diagnosis/types/.

[3] Misaghi A, Goldin A, Awad M, Kulidjian AA (2018). Osteosarcoma: a comprehensive review. SICOT J.

[4] Ritter J, Bielack SS (2010). Osteosarcoma. Ann Oncol.

[5] Mirabello L, Zhu B, Koster R (2020). Frequency of pathogenic germline variants in cancer-susceptibility genes in patients with osteosarcoma. JAMA Oncol.

[6] St. Jude Children's Research Hospital (2023). Osteosarcoma - St. Jude Children's Research Hospital. Osteosarcoma [Internet]. Memphis, TN: St. Jude Children's Research.

[7] Johnstone C, Lutz ST (2014). External beam radiotherapy and bone metastases. Ann Palliat Med.

[8] (2023). External beam radiation therapy (EBRT). Duarte, CA: City of Hope.

[9] Odri GA, Tchicaya-Bouanga J, Yoon DJ, Modrowski D (2022). Metastatic progression of osteosarcomas: a review of current knowledge of environmental versus oncogenic drivers. Cancers (Basel).

[10] (2023). The Science Behind Radiation Therapy. American Cancer Society. The Science Behind Radiation Therapy [Internet.

[11] (2023). Blood tests for bone cancer. https://www.cancerresearchuk.org/about-cancer/bone-cancer/getting-diagnosed/tests-for-bone-cancer/blood-tests.

[12] Syed R, Bomanji J, Nagabhushan N (2006). 186Re-HEDP in the treatment of patients with inoperable osteosarcoma. J Nucl Med.

[13] Papandreou C, Skopelitou A, Kappes G, Daouaher H (2010). Primary osteosarcoma of the urinary bladder treated with external radiotherapy in a patient with a history of transitional cell carcinoma: a case report. J Med Case Rep.

[14] Morris B, Williams W, Shuttleworth GN (2006). Osteosarcoma after external beam radiation therapy for recurrent choroidal melanoma. Ophthalmic Plast Reconstr Surg.

[15] Machak GN, Tkachev SI, Solovyev YN (2003). Neoadjuvant chemotherapy and local radiotherapy for high-grade osteosarcoma of the extremities. Mayo Clin Proc.

[16] Kallianpur AA, Gupta R, Muduly DK, Kapali A, Subbarao KC (2013). Osteosarcoma of breast: a rare case of extraskeletal osteosarcoma. J Cancer Res Ther.

[17] Jiménez I, Laé M, Tanguy ML (2020). Craniofacial second primary tumors in patients with germline retinoblastoma previously treated with external beam radiotherapy: a retrospective institutional analysis. Pediatr Blood Cancer.

[18] Imhof SM, Moll AC, Hofman P, Mourits MP, Schipper J, Tan KE (1997). Second primary tumours in hereditary- and nonhereditary retinoblastoma patients treated with megavoltage external beam irradiation. Doc Ophthalmol.

[19] Douglas YL, Meuzelaar KJ, van der Lei B (1997). Osteosarcoma of the sternum. Eur J Surg Oncol.

[20] DeLaney TF, Park L, Goldberg SI, Hug EB, Liebsch NJ, Munzenrider JE, Suit HD (2005). Radiotherapy for local control of osteosarcoma. Int J Radiat Oncol Biol Phys.

[21] Couldwell WT, Scheithauer BW, Rice SG, Zhang W, Stillerman CB (1997). Osteosarcoma of the meninges in association with glioblastoma. Acta Neurochir (Wien).

[22] Bhatt NR, Kakked GA, Merchant R, Bhatt R (2014). Extraskeletal osteosarcoma of the larynx: an extremely unusual tumour. BMJ Case Rep.

[23] Anderson P, Nuñez R (2007). Samarium lexidronam (153Sm-EDTMP): skeletal radiation for osteoblastic bone metastases and osteosarcoma. Expert Rev Anticancer Ther.

[24] Martí-Bonmatí L, Sopena R, Bartumeus P, Sopena P (2010). Multimodality imaging techniques. Contrast Media Mol Imaging.

[25] Daly T (2020). Evolution of definitive external beam radiation therapy in the treatment of prostate cancer. World J Urol.

[26] Hristov B, Shokek O, Frassica DA (2007). The role of radiation treatment in the contemporary management of bone tumors. J Natl Compr Canc Netw.

[27] Ríos I, Vásquez I, Cuervo E, Garzón Ó, Burbano J (2018). Problems and solutions in IGRT for cervical cancer. Rep Pract Oncol Radiother.

[28] Smith RA, Andrews KS, Brooks D, Fedewa SA, Manassaram-Baptiste D, Saslow D, Wender RC (2019). Cancer screening in the United States, 2019: a review of current American Cancer Society guidelines and current issues in cancer screening. CA Cancer J Clin.

